# The utility of standardized advance directives: the general practitioners’ perspective

**DOI:** 10.1007/s11019-016-9688-3

**Published:** 2016-02-09

**Authors:** Ina Carola Otte, Bernice Elger, Corinna Jung, Klaus Walter Bally

**Affiliations:** Institute of Biomedical Ethics, University of Basel, Basel, Switzerland; Institute of Primary Health Care, University of Basel, Basel, Switzerland; Facharzt für Allgemeine Medizin FMH, Universitäres Zentrum für Hausarztmedizin beider Basel, St. Johanns-Parkweg 2, 4056 Basel, Switzerland

**Keywords:** Advance care planning, Advance directives, Ethics, General practice, General practitioners, Primary care, Templates

## Abstract

Advance directives (AD) are written documents that give patients the opportunity to communicate their preferences regarding treatments they do or do not want to receive in case they become unable to make decisions. Commonly used pre-printed forms have different formats. Some offer space for patients to (a) appoint a surrogate decision maker, and/or (b) to determine future medical treatments and/or (c) give a statement of personal values. So far it is unknown which forms GPs preferably use and why they decide to do so. 23 semi-structured interviews with GPs were analysed using content analysis. Interviewees mainly use short templates (to appoint surrogate decision makers) and medium length templates with checkboxes to indicate patients’ preferences in regards to life prolonging measures. Especially when patients faced the progression of a disease, participants use the latter version. Only then, the interviewees remarked, patients are capable to rate concrete situations reliably. GPs also realize the importance of the verbal assessment of patients’ preferences; however they rarely keep a written form of the conversation. Some GPs hand out one or more templates and ask their patients to read and think about them at home with the option to talk to them about it later on, while others prefer their patients to fill them out alone at home. Regardless of template usage, most GPs emphasize that ADs require regular updates. GPs tend to see standardized advance directives mainly as a tool to start a conversation with their patients and to identify their real preferences and values. When the patient is still not facing the progression of an already existing disease it could be sufficient to only appoint a surrogate decision maker instead of creating a full AD. However, in all other situations, appointing a surrogate decision maker should be backed up by a written statement of a patient’s general values. Patients and their relatives should always have the opportunity to ask their GP for medical advice when drafting an AD. It is crucial to regularly verify and update existing ADs within the course of a disease.

## Introduction

Advance directives (AD) are written documents that give patients the opportunity to communicate their preferences regarding treatments they do or do not want to receive in case they become unable to make decisions. ADs also often offer space for patients to (a) appoint a surrogate decision maker, (b) determine future medical treatments and/or (c) give a personal statement. ADs are most useful for treating physicians, patients, and their relatives when they are drafted in a clear, understandable, and not too broad manner, simultaneously covering all important and relevant medical information (Johnson et al. 2015).

The process to draft such an AD can be very time-consuming, especially for older patients (Lum et al. 2015). Nevertheless, general practitioners (GPs) are good candidates to achieve the desired level of quality in ADs since they often treat their patients in a holistic way and for an extended period of time (Leal Hernandez et al. 2015; Harringer 2012). In practice however, studies show that GPs often use standardized forms assisting patients in drawing up an AD. Unfortunately, individual statements of any given patient rarely are added to these forms (Harringer 2012; Nauck et al. 2014). The existing body of literature highlights several limitations of ADs that are linked to this phenomenon. Short forms (often used only to appoint a surrogate decision maker) increase the likelihood of excluding patients’ wishes to prolong or determine therapy (Shalowitz et al. 2006; Coppolino and Ackerson 2001). Furthermore, Pautex et al. (2015) found, that patients expect their relatives to play an active role in future medical decision making, but often do not share specific wishes and preferences with them.

Also, the accuracy of healthy patients’ stated preferences regarding future treatment choices varies (Nauck et al. 2014; Shalowitz et al. 2006). The content, structure, and underlying attitude of standard forms can influence patients, depending on their source; for example, forms provided by the Catholic Church as compared to those from ‘right-to-die’ organizations (Nauck et al. 2014; Kressel et al. 2007).

Studies also show that the practical use of these templates is often limited by various factors, such as the usage of statements that are too general in nature (Voltz et al. 1998; Porensky and Carpenter 2008; Burchardi et al. 2005; Hobbs 2007).

In Switzerland, the 2013 adult protection law strengthens the legal status of ADs. Different organisations [for example the Swiss Medical Association FMH, Caritas, ethics consultation services (Dialog Ethik) as well as right-to-die- organizations (Exit)] offer pre-printed templates to facilitate the process of generating an AD. In general, three different types of templates are currently in use. The shortest version of the available templates only allows patients to appoint a person as a surrogate decision maker (Evans et al. 2012; Robertson 1995). The version of medium length uses checkboxes to assess patients’ values with a special focus on life-sustaining treatments and the discontinuation of therapy. The longest version offers additional space to write individual statements.

GPs often emphasize the importance of individual anamneses of a patient’s values (Otte et al. 2014) and patients often come to their GP to draft an AD (Ashby et al. 1995). We therefore seek to better understand the process of drawing up ADs in general practice. Up until now, no qualitative data exists, explaining which standardized templates GPs use and for what reasons.

Our qualitative study is the first to elaborate on whether GPs use standardized templates, and if so, which version, why, and whether they change/add a patient’s individual explanations to the forms. Our study also sheds light on potential concerns and shortcomings related to the use of templates as well as barriers GPs encounter when assisting their patients in drawing up ADs.

## Methods

This paper references results from a nation-wide study entitled “Conditions and Quality of End-of-Life Care in Switzerland—the role of general practitioners,” which was funded by the Swiss National Science Foundation. The aim of this study is to conduct a detailed exploration of the functions of general practitioners, who administer palliative care in primary practice. Twenty-three qualitative interviews with general practitioners were conducted and analysed.

### Sampling and data collection

To obtain a diverse selection of physicians working in different practice settings (group versus single), regions (different cantons, rural versus urban region etc.), gender, age, and professional experience, 30 general practitioners were purposively selected from the FMH (Swiss Medical Association) list (proportional quota sampling). Participants were contacted via e-mail outlining the research. Of this sample, 23 physicians agreed to participate (positive respond rate of 76 %). In a 1 h face-to-face interview in their practices, participants answered questions about administering palliative care in a primary care setting. No one other than the interviewers and the interviewee was present during the interview. The qualitative semi-structured interview guideline consisted of 20 questions. The interviewer started with a broader question about the importance of ADs in GPs daily work. Then the GP was asked, which patients are usually interested in drafting an AD and about GPs experiences how ADs are usually drafted? If a GP mentioned the use of a template he was asked which template and why. Also the GP was asked, who (the patient or the GP) usually starts a conversation about ADs and what GPs feel to be the best moment to start talking about the topic of ADs. The interviews were recorded from December 2012 to February 2013. Participants were asked about administering palliative care, their networking with other institutions and stakeholders, and the meaning of ADs for their work. Additional questions explored when and how they approached this latter topic with their patients. The interview guide was pilot tested and subsequently adapted during the first interviews. Either IO or CJ conducted the interviews in German. Both are sociologists who have long term experience with qualitative methods. An additional interviewer (with training in qualitative methods) was hired to conduct the French interviews. All interviews were transcribed verbatim in the original language of the interviewees (French and several Swiss German dialects) and were analysed with the support of the analysis programme atlas.ti, Version 7.0. Participants were given the opportunity to review their interview transcripts. However, no participant made use of this option.

### Analysis

All four authors (of varying disciplinary background: sociology, general practice, palliative care) analysed the anonymised transcripts. Everyone followed Mayring’s nine steps of content analysis (Mayring 2015): (1) relevant data was defined, (2) context and appearance of the data was registered, (3) a formal characterization of the data material was described, (4) course of analysis was specified, (5) a theory-lead differentiation was checked, (6) technique of analysis was defined (summarization, explication, structuring), (7) unit of analysis was defined, (8) data material was analysed, and (9) finally interpreted. The data was repeatedly coded, moving from concrete passages to a more abstract level of coding. Themes were derived from the data found in repeating concepts. In team meetings, all findings were critically tested and discussed by all coders. Any disagreements were solved by discussion. Theoretical saturation was reached when interviewees repeated prior findings and did not add anything of new significance.

The study was approved by Basel Ethics Committee (Nr. EK 248/12) prior to its initiation. All participants provided their informed consent.

## Results

When questioned about the role of ADs in their daily work, the great majority of those interviewed in our study agree that an AD is of high importance and very useful for their work. According to them, more and more patients ask for assistance in making an AD. GPs frequently report finding ADs to be important because they make it possible to start a conversation about end of life (EOL) issues with their patients. Interviewees often use standardized AD forms that vary in length. The shortest form offers the possibility to appoint a surrogate decision maker while the longest form, additionally offers space for an individual statement from the patient. Templates are mainly completed by the patient at home, without the GP.

### Why do Swiss GPs use standardized AD forms?

The long duration of time with which Swiss GPs see their patients not only serves as the foundation of trust in the relationship, but can at times be a communication barrier. Some interviewees found it difficult to initiate conversations about emotional and sensitive topics with their patients, especially when the patients already suffer from a severe illness:

[GP 2]: The patient was mentally always very fit and I knew him for quite a while. I really had some doubts and constraints talking to him about ADs

According to interviewees, the available templates are a helpful instrument for them to start conversations on end of life questions whereby the GP can then assess wishes and values of patients:

[GP 7]: So, to me it (the AD) is basically a starter and a reminder that we have discussed this topic with the patients. It gives me a frame and room to talk about it; it (the template) is something official that I can use to talk about these topics
[GP 6]: So, I use it to verbally explore patients’ wishes and having a short template makes leading such a conversation much easier for me
[GP 8]: Advance directives are helpful to start a conversation not only between doctor and patient but also between patient and involved relatives. It is also easier for me to assess a patient’s values during the conversation, where the patient has the possibility to ask questions; so I can get a feeling for a patient’s values. So I think I actually use it more as an instrument to verbally assess patient’s future treatment wishes. For me, having a template for advance directives makes the start of this conversation much easier.

### How begins a conversation about ADs und what kinds of templates are used?

The way a GP starts a conversation on the topic of ADs can influence the quality of it. Some GPs hand out one or more templates and ask their patients to read and think about them at home with the option to talk to them about it later on, while others prefer their patients to fill them out alone at home. Other GPs assess and document patients’ preferences in more than one consultation, sometimes even together with a patient’s relatives.

[GP 5]: Patients sometimes ask me, “Do you have an AD that I can sign?” and I do have different templates, which I hand out in this case. I tell them to think about the content, do they feel that it covers all important topics? Or do they disagree with some parts? And if they feel the template suits them, we usually sign it together the next time we see each other
[GP 1]: I cannot tell my patients what to do or what not to do, I tell them their possibilities and options and then I tell them to discuss it with their family and to document it as good as possible, what they would want later on and what not.

1. Short standardized AD forms are mainly used to appoint a surrogate decision maker.

Some of the interviewees use the shortest version of the available templates mainly to name a surrogate decision maker, since they find it difficult for patients to anticipate future hypothetical scenarios. Another reason participants choose to use the shortest version is to avoid the burden of talking about “terminal scenarios.”

[GP 8]: My experience is that most of my patients prefer the shortest version possible, where they only name a surrogate decision maker
[GP 4]: …and that is the thing, you cannot cover every eventuality in an AD that is way too complicated and impossible to achieve. And as slong as you’re healthy you simply don’t need a special document, you can simply take the short official version of the Swiss Medical Association FMH. But as soon as things change, you need to reconsider this choice
[GP 9]: So, I use 2 templates, the shorter one (to name a surrogate decision maker) and the longer version, which I only offer if the patients explicitly want it. But these scenarios that the longer version includes are so hypothetical; in my opinion it barely makes sense to use that one
[GP 7]: In general, using an AD with the intent to alleviate pain is in my opinion okay; however, talking about PEG-tubes, enteral nutrition and resuscitation with my patient is difficult for me. I mainly use it to appoint a surrogate decision maker

GP 11 uses the shortest form because the template is only a tool for him to start a conversation in which he can assess a patient’s wishes, needs, and values.

[GP 11]: The paper is not important, it is important that you get to know the patient. So I use the shortest version as a starter, but the real assessment of patients’ wishes takes place during the conversation we have.

2. Standardized AD templates, which offer the possibility for the patient to express his/her wishes regarding future life-prolonging treatments.

A few participants use the medium length version in which patients can, in addition to naming a surrogate decision maker, express their wishes regarding life prolonging treatments by ticking off checkboxes. However, GPs tend to refrain from offering these templates to healthy patients. Instead, GPs mainly offer versions that provide patients the possibility to either accept or decline the use of a PEG tube or antibiotics and/or resuscitation to those with a serious condition.

Their reason for this trend is that GPs often fear that patients cannot realistically imagine future scenarios, such as swallowing inability after a stroke. The more detailed versions allow GPs to address concrete situations and to add changes to the AD if necessary.

[GP 11]: And if I fill in an AD with my patients, I always advise them to make a lot of changes to the form, because especially the medium form includes so many situations that are highly hypothetical and very abstract, it does not make any sense to fill it in
[GP 10]: Well, so there is a form from the FMH, it is very short and here is a longer form. So the medium one, I always use that for the patients, but I find these situations very broad and often too far away from reality, so I see no sense in that
[GP 8]: As long as a patient is still healthy, the short form is sufficient, but as soon as a serious illness progresses it is useful to have a longer version which determines if in case of a pneumonia he/she’d like to receive antibiotics or not.

3. Standardized templates paired with individual anamneses:

Many GPs emphasize the importance of assessing individual values of their patients in order to correctly ascertain their future treatment wishes. However, participants are not definitive as to whether they also keep a written form of these additional individual anamneses.

[GP 3]: Sometimes patients ask me why they should have an AD. Why is it important to have one? And then I tell them that it is important for me, so we can talk about it and I can understand their thoughts on different things and so I know what they would want in case they cannot express their wishes any longer
[GP 6]: So ADs are a tool for a patient to express his/her wishes in situations where he/she is left unable to communicate them. But they also help me before these situations occur, because I get the possibility to talk about potential questions and therefore assess a patient’s values and wishes. So it is a great tool which makes it easy to start this conversation and to assess his/her values during it
[GP 9]: For me it is important to get to know the thoughts of a patient. What are his/her attitudes regarding different options and how are his/her attitudes/preferences different than mine? So we can find a compromise, something we agree on
[GP 19]: All right, well I am going to be very provocative. Advance directives are very useful when we discuss them with the patient… Advance directives are an issue where I am going to take 1 or 2 h and sometimes more than one conversation, in order to discuss this topic with my patients … I do it with sick patients in order to find out what are their wishes and in order to try to understand how (.) they imagine future things. However, the problem with advance directives is, when we do it with patients who are still healthy, is that they are not able to imagine future illness

Regardless of the template GPs use, most emphasize that ADs are legally binding documents, which require regular updates.

[GP 3]: I find it important that my patients start thinking about questions related to future treatment decisions and I also have to say since ADs are a legally binding document we have to update them regularly, so they are still valid later on … but the process of thinking about topics related to ADs is something, that I always support in my patients
[GP 6]: Well, as soon as a patient’s situation changes, I man, maybe a disease that is progressing or cancer and the prognosis changes as well, then we could adapt the existing AD to make it fit the new situation. And that is the main topic to me, as soon as someone becomes seriously ill, … “We made an AD 2 years ago, what do you think, what would you like in case for example you’re suffering from dyspnea? Should we give you antibiotics in case of an pneumonia or just morphine? We need to talk about these things, no?”

## Discussion

For most GPs in this study, ADs are an important tool to start a conversation about difficult topics, such as approaching death or death itself. According to the participants of this study, the assessment of the personal values of the patient during this conversation weighs more than the written AD in the end. Often they use either the short (only surrogate decision maker) or the medium length version (surrogate decision maker and check boxes concerning future medical treatment) of the available templates. However, most feel that the situations described in the latter version are highly hypothetical. Although interviewed GPs mentioned the existence of the longest version, which consists of an individually written statement, they do not actively use it.

For the medium length version, interviewees stated concerns that, in their opinion, pre-printed forms are too hypothetical to cover all important aspects and therefore offer space for misunderstandings and misinterpretation. This fear of misunderstandings is in line with results of a qualitative study by Thompson et al., where participant hospital physicians and nursing professionals note the possible negative effects of advance directives, such as the risk of misinterpretation or general errors in treatment (under- or paradoxical overtreatment) (Thompson et al. 2003). Nevertheless, Harringer’s study from (2012) shows that GPs—despite all concerns—often use short to medium length forms for ADs. Some of our interviewees explicitly claim that it is not the form, but the conversation itself that matters to them. Under this approach, the final document (which they think is too hypothetical anyway) seems to lose some of its importance, while the focus clearly lies on the verbal anamneses of patient’s values. Emanuel et al. (1995) note the importance of verbal anamneses. Recent studies elaborate on other efforts to further support patients in light of the common issues experienced during end of life scenarios in non-confrontational settings. For example, different approaches can be utilized in conjunction with the go wish card game (Lankarani-Fard et al. 2010).

Furthermore, the guidelines of the SAMS (Swiss Academy of Medical Sciences) regarding medical communication indicate the low value of short and standardized advance directives (Schweizerische Akademie der Medizinischen Wissenschaften 2013). Based on these guidelines, standardized advance directives cannot express individual values because standardized sentences cannot sufficiently illustrate a patient’s health and/or biographical background (Schweizerische Akademie der Medizinischen Wissenschaften 2013; Lack 2008). Broad statements such as wanting to “maintain dignity” or be “free from pain”, are often too general to provide a basis for individual treatment decisions (Coppola et al. 2001; Lo and Steinbrook 2004). For example, ADs often refer to forgoing an intervention when the patient’s condition is “irreversible” or “terminal”. However, determining whether patients are in these states is often very difficult (Sudore and Fried 2010).

Additionally, broad statements increase the possibility of contradictions with patients’ stated wishes (Lack 2008). Individuality of an AD is therefore often considered as one of the main indicators of quality (Lack 2008). Patients’ treatment preferences and values change as their health changes (Halpern and Arnold 2008; Loewenstein 2005; Koch 2001), at the end of life (Fried et al. 2007), and even during periods of stable health (Fried et al. 2007). GPs in this study shared their concern over making ADs with patients who are still healthy because they fear patients would not be able to consistently anticipate future scenarios and treatment preferences. This is in line with the results of Bauer (2009).

Since an AD is a legal document, stated wishes must be as authentic as possible and include specific wording to avoid possible misunderstandings. Regular updates are therefore of the utmost importance. The Swiss Academy of Medical Sciences (SAMS) guidelines recommend that ADs should be part of a process, which spans more than one conversation to assess and update values, treatment goals, and possible proxies. However, our interviewees rarely mentioned updates. Interviewees reported offering follow up conversations (as recommended by the SAMS) only when the patient actively asked for it.

Interviewed GPs use the shortest template mainly to start a conversation and appoint surrogate decision makers. However, when the appointment of a surrogate is often the only written document in the end, it is questionable whether the surrogate’s decisions are congruent with the patient’s wishes. Shalowitz et al. (2006) found that patient-designated surrogates incorrectly predict patients’ end-of-life treatment preferences in one third of cases. Also patients’ fears of abuse by relatives and possible surrogates is a factor that can limit the employment of an AD (Sahm et al. 2005). Following the example set by one of the GPs (GP 8), it can be sufficient to use only the short form to appoint a surrogate decision maker during a patient’s healthy days; however, as soon as a serious disease progresses, a more detailed version should be used to offer the patient space to document personal values and preferences as well as anticipated decisions for the future (Evans et al. 2012).

According to our results, patients often complete an AD template at home, without the support of their GP. This can lead to various difficulties and inaccuracies that can compromise the quality of an AD (Nauck et al. 2014; Spoelhof and Elliott 2012). In order to avoid possible inaccuracies, drafting a useful AD usually takes a few consultations, which also provides the patient time to ask questions.

Further, since there is a pool of different forms for ADs from which to draw (from religious organizations as well as from right-to-die organizations), the one a GP hands out can insinuate certain choices for the patient and therefore influence the patients’ follow up decisions. Moreover, while the idea to elaborate patient’s values during the conversation could be a good start for assessment, the information a patient receives might not be sufficient for him/her to complete an AD at home. Patients often feel ambivalent about different treatment options and therefore need support and help with their decision making (Frost et al. 2011). Modifiable factors such as knowledge gaps, uncertainty regarding outcomes, lack of clarity about what matters most, and feeling pressured to choose a particular option may exacerbate the decisional conflict and make the support of the treating GP essential (Murray et al. 2009).

## Conclusions

Standardized advance directives are important tools for GPs and offer a good basis for them to start a conversation about patients’ preferences and future treatment wishes.

When the patient is still not facing the progression of an already existing disease it could be sufficient to only appoint a surrogate decision maker instead of creating a full AD, since preferences often change during the course of illness.

However, in all other situations, the appointing of a surrogate decision maker should be supported with a written statement of the patient’s general values. In order to avoid broad and general statements, tools such as the go wish card game with a variety of pre-formulated value attitudes could be helpful.

Patients and their relatives should always have the opportunity to ask their GP for medical advice when drafting an AD.

It is crucial to regularly verify and update existing ADs within the course of a disease.

## Strengths and limitations

A clear strength of this study is the use of a qualitative method to explore a multifaceted topic, in which GPs could express how they integrate advance directives in their practice.

Since our study is a qualitative study we are not able to reach a conclusion regarding the quantitative aspects and distributions of opinions among GPs.

Furthermore, because our results rely solely on qualitative data, triangulation from other methods of data collection, such as a survey, may increase the validity of the results. For this reason, the next step of our study is a large-scale questionnaire to quantify the results that we obtained from our interviews.

## Abbreviations

GP(s): General practitioner(s)
AD(s): Advance directive(s)

## References

[CR1] Ashby M, Wakefield M, Beilby J (1995). General practitioners’ knowledge and use of living wills. BMJ.

[CR2] Bauer AW (2009). Chances and limitations of patients’ advance decisions at the end of life. Wiener Medizinische Wochenschrift.

[CR3] Burchardi N, Rauprich O, Hecht M, Beck M, Vollmann J (2005). Discussing living wills. A qualitative study of a German sample of neurologists and ALS patients. Journal of the Neurological Sciences.

[CR4] Coppola KM, Ditto PH, Danks JH, Smucker WD (2001). Accuracy of primary care and hospital- based physicians’ predictions of elderly outpatients’ treatment preferences with and without advance directives. Archives of Internal Medicine.

[CR5] Coppolino M, Ackerson L (2001). Do surrogate decision makers provide accurate consent for intensive care research?. Chest.

[CR6] Emanuel LL, Danis M, Pearlman RA, Singer PA (1995). Advance care planning as a process: Structuring the discussions in practice. Journal of the American Geriatrics Society.

[CR7] Evans N, Bausewein C, Menaca A, Andrew EV, Higginson IJ, Harding R (2012). A critical review of advance directives in Germany: Attitudes, use and healthcare professionals’ compliance. Patient Education and Counseling.

[CR8] Fried TR, O’Leary J, Van Ness P, Fraenkel L (2007). Inconsistency over time in the preferences of older persons with advanced illness for life-sustaining treatment. Journal of the American Geriatrics Society.

[CR9] Frost DW, Cook DJ, Heyland DK, Fowler RA (2011). Patient and healthcare professional factors influencing end-of-life decision-making during critical illness: A systematic review. Critical Care Medicine.

[CR10] Halpern J, Arnold RM (2008). Affective forecasting: An unrecognized challenge in making serious health decisions. Journal of General Internal Medicine.

[CR11] Harringer W (2012). Advance directives in general practice. Therapeutische Umschau Revue therapeutique.

[CR12] Hobbs P (2007). The limitations of advance directives and statements in mental health. Australasian Psychiatry: Bulletin of Royal Australian and New Zealand College of Psychiatrists.

[CR13] Johnson CE, Singer R, Masso M, Sellars M, Silvester W (2015). Palliative care health professionals’ experiences of caring for patients with advance care directives. Australian Health Review: a publication of the Australian Hospital Association.

[CR14] Koch T (2001). Future states: The axioms underlying prospective, future-oriented, health planning instruments. Social Science and Medicine.

[CR15] Kressel LM, Chapman GB, Leventhal E (2007). The influence of default options on the expression of end-of-life treatment preferences in advance directives. Journal of General Internal Medicine.

[CR16] Lack P (2008). Different forms of patient advance directives and their importance to particular groups of people. Bulletin de la Societe des Sciences Medicales du Grand-Duche de Luxembourg.

[CR17] Lankarani-Fard A, Knapp H, Lorenz KA, Golden JF, Taylor A, Feld JE (2010). Feasibility of discussing end-of-life care goals with inpatients using a structured, conversational approach: The go wish card game. Journal of Pain and Symptom Management.

[CR18] Leal Hernandez M, RivasBaez JA, MartinezMonje F, LozanoEspinosa M (2015). Role of the family doctor in the completion and registration of advance directives documents. Semergen/Sociedad Espanola de Medicina Rural y Generalista.

[CR19] Lo B, Steinbrook R (2004). Resuscitating advance directives. Archives of Internal Medicine.

[CR20] Loewenstein G (2005). Projection bias in medical decision making. Medical Decision Making: An International Journal of the Society for Medical Decision Making.

[CR21] Lum HD, Sudore RL, Bekelman DB (2015). Advance care planning in the elderly. The Medical Clinics of North America.

[CR26] Mayring Philipp (2015). Qualitative Inhaltsanalyse: Grundlagen und Techniken.

[CR22] Murray MA, Brunier G, Chung JO, Craig LA, Mills C, Thomas A (2009). A systematic review of factors influencing decision-making in adults living with chronic kidney disease. Patient Education and Counseling.

[CR23] Nauck F, Becker M, King C, Radbruch L, Voltz R, Jaspers B (2014). To what extent are the wishes of a signatory reflected in their advance directive: A qualitative analysis. BMC Medical Ethics.

[CR24] Otte IC, Jung C, Elger BS, Bally K (2014). Advance directives and the impact of timing. A qualitative study with Swiss general practitioners. Swiss Medical Weekly.

[CR25] Pautex S, Gamondi C, Philippin Y, Gremaud G, Herrmann F, Camartin C, Vayne-Bossert P (2015). Advance directives and end-of-life decisions in Switzerland: Role of patients, relatives and health professionals. BMJ Support Palliat Care.

[CR27] Porensky EK, Carpenter BD (2008). Knowledge and perceptions in advance care planning. Journal of Aging and Health.

[CR28] Robertson GS (1995). Making an advance directive. BMJ.

[CR29] Sahm S, Will R, Hommel G (2005). Attitudes towards and barriers to writing advance directives amongst cancer patients, healthy controls, and medical staff. Journal of Medical Ethics.

[CR30] Schweizerische Akademie der Medizinischen Wissenschaften (SAMW). 2013. Kommunikation im medizinischen Alltag: Ein Leitfaden für die Praxis.

[CR31] Shalowitz DI, Garrett-Mayer E, Wendler D (2006). The accuracy of surrogate decision makers: A systematic review. Archives of Internal Medicine.

[CR32] Spoelhof GD, Elliott B (2012). Implementing advance directives in office practice. American Family Physician.

[CR33] Sudore RL, Fried TR (2010). Redefining the “planning” in advance care planning: Preparing for end-of-life decision making. Annals of Internal Medicine.

[CR34] Thompson TD, Barbour RS, Schwartz L (2003). Health professionals’ views on advance directives: A qualitative interdisciplinary study. Palliative Medicine.

[CR35] Voltz R, Akabayashi A, Reese C, Ohi G, Sass HM (1998). End-of-life decisions and advance directives in palliative care: A cross-cultural survey of patients and health-care professionals. Journal of Pain and Symptom Management.

